# Probability Mass Exclusions and the Directed Components of Mutual Information

**DOI:** 10.3390/e20110826

**Published:** 2018-10-28

**Authors:** Conor Finn, Joseph T. Lizier

**Affiliations:** 1 Complex Systems Research Group and Centre for Complex Systems, Faculty of Engineering & IT, The University of Sydney, NSW 2006, Australia; 2 CSIRO Data61, Marsfield, NSW 2122, Australia

**Keywords:** entropy, mutual information, pointwise, directed, information decomposition

## Abstract

Information is often described as a reduction of uncertainty associated with a restriction of possible choices. Despite appearing in Hartley’s foundational work on information theory, there is a surprising lack of a formal treatment of this interpretation in terms of exclusions. This paper addresses the gap by providing an explicit characterisation of information in terms of probability mass exclusions. It then demonstrates that different exclusions can yield the same amount of information and discusses the insight this provides about how information is shared amongst random variables—lack of progress in this area is a key barrier preventing us from understanding how information is distributed in complex systems. The paper closes by deriving a decomposition of the mutual information which can distinguish between differing exclusions; this provides surprising insight into the nature of directed information.

## 1. Introduction

In information theory, there is a duality between the concepts entropy and information: entropy is a measure of uncertainty or freedom of choice, whereas information is a measure of reduction of uncertainty (increase in certainty) or restriction of choice. Interestingly, this description of information as a restriction of choice predates even Shannon [1], originating with Hartley [2]:

“By successive selections a sequence of symbols is brought to the listener’s attention. At each selection there are eliminated all of the other symbols which might have been chosen. As the selections proceed more and more possible symbol sequences are eliminated, and we say that the information becomes more precise.”

Indeed, this interpretation led Hartley to derive the measure of information associated with a set of equally likely choices, which Shannon later generalised to account for unequally likely choices. Nevertheless, despite being used since the foundation of information theory, there is a surprising lack of a formal characterisation of information in terms of the elimination of choice. Both Fano [3] and Ash [4] motivate the notion of information in this way, but go on to derive the measure without explicit reference to the restriction of choice. More specifically, their motivational examples consider a set of possible choices X modelled by a random variable *X*. Then in alignment with Hartley’s description, they consider information to be something which excludes possible choices *x*, with more eliminations corresponding to greater information; however, this approach does not capture the concept of information in its most general sense since it cannot account for information provided by partial eliminations which merely reduces the likelihood of a choice *x* from occurring. (Of course, despite motivating the notion of information in this way, both Fano and Ash provide Shannon’s generalised measure of information which can account for unequally likely choices.) Nonetheless, Section 2 of this paper generalises Hartley’s interpretation of information by providing a formal characterisation of information in terms of probability mass exclusions.

Our interest in providing a formal interpretation of information in terms of exclusions is driven by a desire to understand how information is distributed in complex systems [5,6]. In particular, we are interested in decomposing the total information provided by a set of source variables about one or more target variables into the following atoms of information: the unique information provided by each individual source variable, the shared information that could be provided by two or more source variables, and the synergistic information which is only available through simultaneous knowledge of two or more variables [7]. This idea was originally proposed by Williams and Beer who also introduced an axiomatic framework for such a decomposition [8]. However, flaws have been identified with a specific detail in their approach regarding “whether different random variables carry *the same* information or just *the same amount* of information” [9] (see also [10,11]). With this problem in mind, Section 3 discusses how probability mass exclusions may provide a principled method for determining if variables provide the same information. Based upon this, Section 4 derives an information-theoretic expression which can distinguish between different probability mass exclusions. Finally, Section 5 closes by discussing how this expression could be used to identify when distinct events provide the same information.

## 2. Information and Eliminations

Consider two random variables *X* and *Y* with discrete sample spaces X and Y, and say that we are trying to predict or infer the value of an event *x* from *X* using an event *y* from *Y* which has occurred jointly. Ideally, there is a one-to-one correspondence between the occurrence of events from *X* and *Y* such that an event *x* can be exactly predicted using an event *y*. However, in most complex systems, the presence of noise or some other such ambiguity means that we typically do not have this ideal correspondence. Nevertheless, when a particular event *y* is observed, knowledge of the distributions P(Y) and P(X,Y) can be utilised to improve the prediction on average by using the posterior P(X|y) in place of the prior P(X). Our goal now is to understand how Hartley’s description relates to the notion of conditional probability.

When a particular event *y* is observed, we know that the complementary event $\bar{y}=\left\{Y\backslash y\right\}$ did not occur. Thus we can consider the joint distribution P(X,Y) and eliminate the probability mass which is associated with this complementary event $\bar{y}$. In other words, we exclude the probability mass $P(X,\bar{y})$ which leaves only the probability mass P(X,y) remaining. The surviving probability mass can then be normalised by dividing by p(y), which, by definition, yields the conditional distribution P(X|y). Hence, with this elimination process in mind, consider the following definition:

**Definition** **1** (Probability Mass Exclusion)**.** *A probability mass exclusion induced by the event y from the random variable Y is the probability mass associated with the complementary event $\bar{y}$, i.e., $p(\bar{y})$.*

Echoing Hartley’s description, it is perhaps tempting to think that the greater the probability mass exclusion $p(\bar{y})$, the greater the information that *y* provides about *x*; however, this is not true in general. To see this, consider the joint event *x* from the random variable *X*. Knowing the event *x* occurred enables us to categorise the probability mass exclusions induced by *y* into two distinct types: the first is the portion of the probability mass exclusion associated with the complementary event $\bar{x}$, i.e., $p(\bar{x},\bar{y})$; while the second is the portion of the exclusion associated with the event *x*, i.e., $p(x,\bar{y})$. Before discussing these distinct types of exclusion, consider the conditional probability of *x* given *y* written in terms of these two categories,

(1) $$p\left(x|y\right)=\frac{p\left(x\right)-p\left(x,\bar{y}\right)}{1-p\left(x,\bar{y}\right)-p\left(\bar{x},\bar{y}\right)}.$$

**Figure 1:**
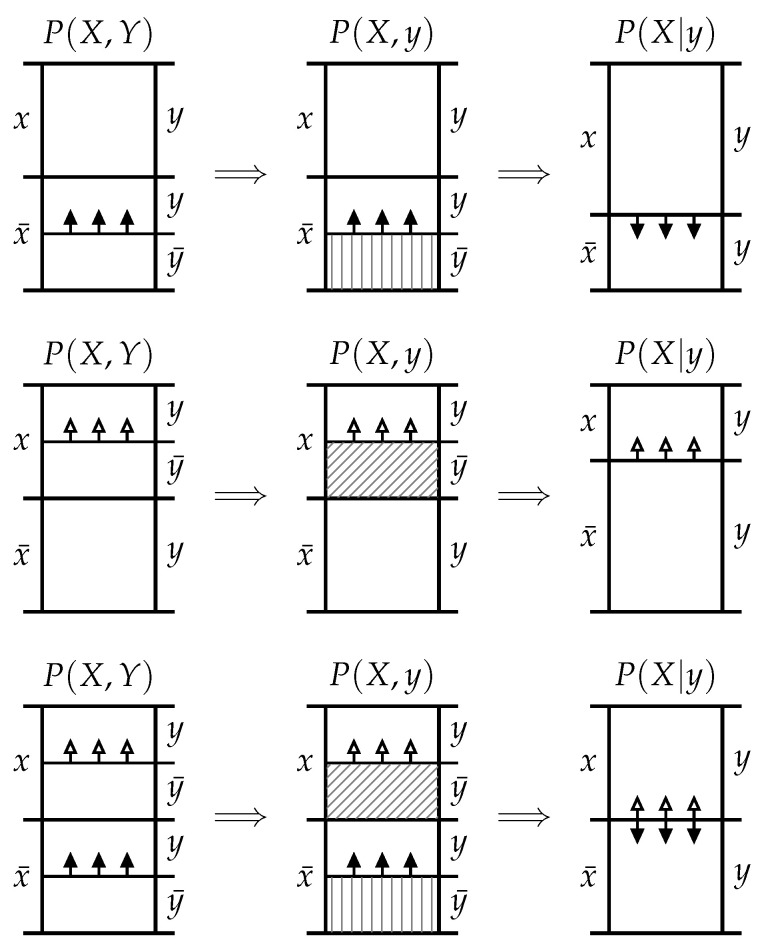
In probability mass diagrams, height represents the probability mass of each joint event from X×Y which must sum to 1. The leftmost of the diagrams depicts the joint distribution P(X,Y), while the central diagrams depict the joint distribution after the occurence of the event y∈Y leads to exclusion of the probability mass associated with the complementary event $\bar{y}$. By convention, vertical and diagonal hatching represent informative and misinformative exclusions, respectively. The rightmost diagrams represent the conditional distribution after the remaining probability mass has been normalised. *Top row*: A purely informative probability mass exclusion, $p(\bar{x},\bar{y})>0$ and $p(x,\bar{y})=0$, leading to p(x|y)>p(x) and hence i(x;y)>0. *Middle row*: A purely misinformative probability mass exclusion, $p(\bar{x},\bar{y})=0$ and $p(x,\bar{y})>0$, leading to p(x|y)<p(x) and hence i(x;y)<0. *Bottom row*: The general case $p(\bar{x},\bar{y}>0)$ and $p(x,\bar{y})>0$. Whether p(x|y) turns out to be greater or less than p(x) depends on the size of both the informative and misinformative exclusions.

To see why these two types of exclusions are distinct, consider two special cases: The first special case is when the event *y* induces exclusions which are confined to the probability mass associated with the complementary event $\bar{x}$. This means that the portion of exclusion $p(\bar{x},\bar{y})$ is non-zero while the portion $p(x,\bar{y})=0$. In this case the posterior p(x|y) is larger than the prior p(x) and is an increasing function of the exclusion $p(\bar{x},\bar{y})$ for a fixed p(x). This can be seen visually in the *probability mass diagram* at the top of Figure 1 or can be formally demonstrated by inserting $p(x,\bar{y})=0$ into (1). In this case, the mutual information

(2) $$i\left(x;y\right)=\log\frac{p(x|y)}{p(x)},$$

is a strictly positive, increasing function of $p(\bar{x},\bar{y})$ for a fixed p(x). (Note that this is the mutual information between events rather than the average mutual information between variables; depending on the context, it is also referred to as the the information density, the pointwise mutual information, or the local mutual information.) For this special case, it is indeed true that the greater the probability mass exclusion $p(\bar{y})$, the greater the information *y* provides about *x*. Hence, we define this type of exclusion as follows:

**Definition** **2** (Informative Probability Mass Exclusion)**.** *For the joint event xy from the random variables X and Y, an informative probability mass exclusion induced by the event y is the portion of the probability mass exclusion associated with the complementary event $\bar{x}$, i.e., $p(\bar{x},\bar{y})$.*

The second special case is when the event *y* induces exclusions which are confined to the probability mass associated with the event *x*. This means that the portion of exclusion $p(\bar{x},\bar{y})=0$ while the potion $p(x,\bar{y})$ is non-zero. In this case, the posterior p(x|y) is smaller than the prior p(x) and is a decreasing function of the exclusion $p(x,\bar{y})$ for a fixed p(x). This can be seen visually in the probability mass diagram in the middle row of Figure 1 or can be formally demonstrated by inserting $p(\bar{x},\bar{y})=0$ into (1). In this case, the mutual information (2) is a strictly negative, decreasing function of $p(x,\bar{y})$ for fixed p(x). (Although the mutual information is non-negative when averaged across events from both variables, it may be negative between pairs of events.) This second special case demonstrates that it is not true that the greater the probability mass exclusion $p(\bar{y})$, the greater the information *y* provides about *x*. Hence, we define this type of exclusion as follows:

**Definition** **3** (Misinformative Probability Mass Exclusion)**.** *For the joint event xy from the random variables X and Y, a misinformative probability mass exclusion induced by the event y is the portion of the probability mass exclusion associated with the event x, i.e., $p(x,\bar{y})$.*

Now consider the general case where both informative and misinformative probability mass exclusions are present simultaneously. It is not immediately clear whether the posterior p(x|y) is larger or smaller than the prior p(x), as this depends on the relative size of the informative and misinformative exclusions. Indeed, for a fixed prior p(x), we can vary the informative exclusion $p(\bar{x},\bar{y})$ whilst still maintaining a fixed posterior p(x|y) by co-varying the misinformative exclusion $p(x,\bar{y})$ appropriately; specifically by choosing

(3) $$p\left(x,\bar{y}\right)=\frac{p\left(x\right)-p\left(x|y\right)\left(1-p(\bar{x},\bar{y})\right)}{1-p(x|y)}.$$

Although it is not immediately clear whether the posterior p(x|y) is larger or smaller than the prior p(x), the general case maintains the same monotonic dependence as the two constituent special cases. Specifically, if we fix p(x) and the misinformative exclusion $p(x,\bar{y})$, then the posterior p(x|y) is an increasing function of the informative exclusion $p(\bar{x},\bar{y})$. On the other hand, if we fix p(x) and the informative exclusion $p(\bar{x},\bar{y})$, then the posterior p(x|y) is a decreasing function of the misinformative exclusion $p(x,\bar{y})$. This can been seen visually in the probability mass diagram at the bottom of Figure 1 or can be formally demonstrated by fixing and varying the appropriate values for each case in (1). Finally, the relationship between the mutual information and the exclusions in this general case can be explored by inserting (1) into (2), which yields

(4) $$i\left(x;y\right)=\log\frac{1-p\left(x,\bar{y}\right)/p\left(x\right)}{1-p\left(x,\bar{y}\right)-p\left(\bar{x},\bar{y}\right)}.$$

If p(x) and the misinformative exclusion $p(x,\bar{y})$ are fixed, then i(x;y) is an increasing function of the informative exclusion $p(\bar{x},\bar{y})$. On the other hand, if p(x) and the informative exclusion $p(\bar{x},\bar{y})$ are fixed, then i(x;y) is a decreasing function of the misinformative exclusion $p(x,\bar{y})$. Finally, if both the informative exclusion $p(\bar{x},\bar{y})$ and misinformative exclusion $p(x,\bar{y})$ are fixed, the i(x;y) is an increasing function of p(x).

Now that a formal relationship between eliminations and information has been established using probability theory, we return to the motivational question—can this understanding of information in terms of exclusions aid in our understanding of how random variables share information?

## 3. Information Decomposition and Probability Mass Exclusions

**Figure 2:**
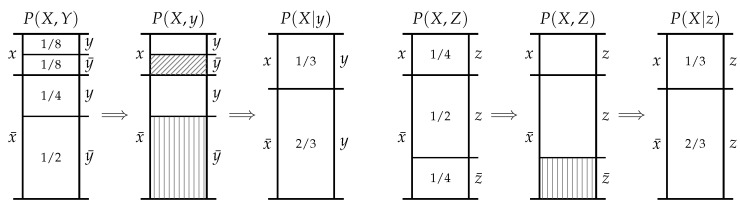
*Top*: probability mass diagram for X×Y. *Bottom*: probability mass diagram for X×Z. Note that the events $y_{1}$ and $z_{1}$ can induce different exclusions in P(X) and yet still yield the same conditional distributions $P\left(X|y_{1}\right)=P\left(X|z_{1}\right)$ and hence provide the same amount of information $i\left(x_{1};y_{1}\right)=i\left(x_{1};z_{1}\right)$ about the event $x_{1}$.

Consider the example in Figure 2 where the events *y* and *z* each induce different exclusions, both in terms of size and type, and yet provide the same amount of information about the event *x* since

(5) i(x;y)=i(x;z)=log4/3≈0.415bit.

The events *y* and *z* reduce our uncertainty about *x* in distinct ways and yet, after making the relevant exclusions, we have the same freedom of choice about *x*. It is our belief that the information provided by *y* and *z* should only be deemed to be the same information if they both reduce our uncertainty about *x* in the same way; we contend that for the events *y* and *z* to reduce our uncertainty about *x* in the same way, they would have to identically restrict our choice, or make the same exclusions with respect to *x*.

What this example demonstrates is that the mutual information does not—and indeed cannot—distinguish between how events provide information about other events. By definition, the mutual information only depends on the prior p(x) and posterior p(x|y) probabilities. Although the posterior p(x|y) depends on both the informative and misinformative exclusions, there is no one-to-one correspondence between these exclusions and the resultant mutual information. Indeed, as we saw in (3), there is a continuous range of informative and misinformative exclusions which could yield any given value for the mutual information. As such, any information decomposition based upon the mutual information alone could never distinguish between how events provide information in terms of exclusions. Thus the question naturally arises—can we express the exclusions in terms of information-theoretic measures such that there is a one-to-one correspondence between exclusions and the measures? Such an expression could be utilised in an information decomposition which can distinguish between whether events provide the same information or merely the same amount of information.

## 4. The Directed Components of Mutual Information

The mutual information cannot distinguish between events which induce different exclusions because any given value could arise from a whole continuum of possible informative and misinformative exclusions. Hence, consider decomposing the mutual information into two separate information-theoretic components. Motivated by the strictly positive mutual information observed in the purely informative case and the strictly negative mutual information observed in the purely informative case, let us demand that one of the components be positive while the other component is negative.

**Postulate** **1** (Decomposition)**.** *The information provided by y about x can be decomposed into two non-negative components, such that $i\left(x;y\right)=i_{+}\left(y\;\to \;x\right)-i_{-}\left(y\;\to \;x\right)$.*

Furthermore, let us demand that the two components preserve the functional dependencies between the mutual information and the informative and misinformative exclusion observed in (4) for the general case.

**Postulate** **2** (Monotonicity)**.** *The functions $i_{+}\left(y\;\to \;x\right)$ and $i_{-}\left(y\;\to \;x\right)$ should satisfy the following conditions:*

*1.*  For all fixed p(x,y) and $p(x,\bar{y})$, the function $i_{+}\left(y\;\to \;x\right)$ is a continuous, increasing function of $p(\bar{x},\bar{y})$.
*2.*  For all fixed $p(\bar{x},y)$ and $p(\bar{x},\bar{y})$, the function $i_{-}\left(y\;\to \;x\right)$ is a continuous, increasing function of $p(x,\bar{y})$.
*3.*  For all fixed p(x,y) and $p(\bar{x},y)$, the functions $i_{+}\left(y\;\to \;x\right)$ and $i_{-}\left(y\;\to \;x\right)$ are increasing and decreasing functions of $p(\bar{x},\bar{y})$, respectively.

Before considering the functions which might satisfy Postulates 1 and 2, there are two further observations to be made about probability mass exclusions. The first observation is that an event *x* could never induce a misinformative exclusion about itself, since the misinformative exclusion $p(x,\bar{x})=0$. Indeed, inserting this result into the self-information in terms of (4) yields the Shannon information content of the event *x*,

(6) $$i\left(x;x\right)=\log\frac{1-p\left(x,\bar{x}\right)/p\left(x\right)}{1-p\left(x,\bar{x}\right)-p\left(\bar{x},\bar{x}\right)}=-\log\left(1-p(\bar{x},\bar{x})\right)=-\log p\left(x\right)=h\left(x\right).$$

**Postulate** **3** (Self-Information)**.** *An event cannot misinform about itself, hence $i_{+}\left(x\;\to \;x\right)=i\left(x;x\right)=h\left(x\right)$.*

**Figure 3:**
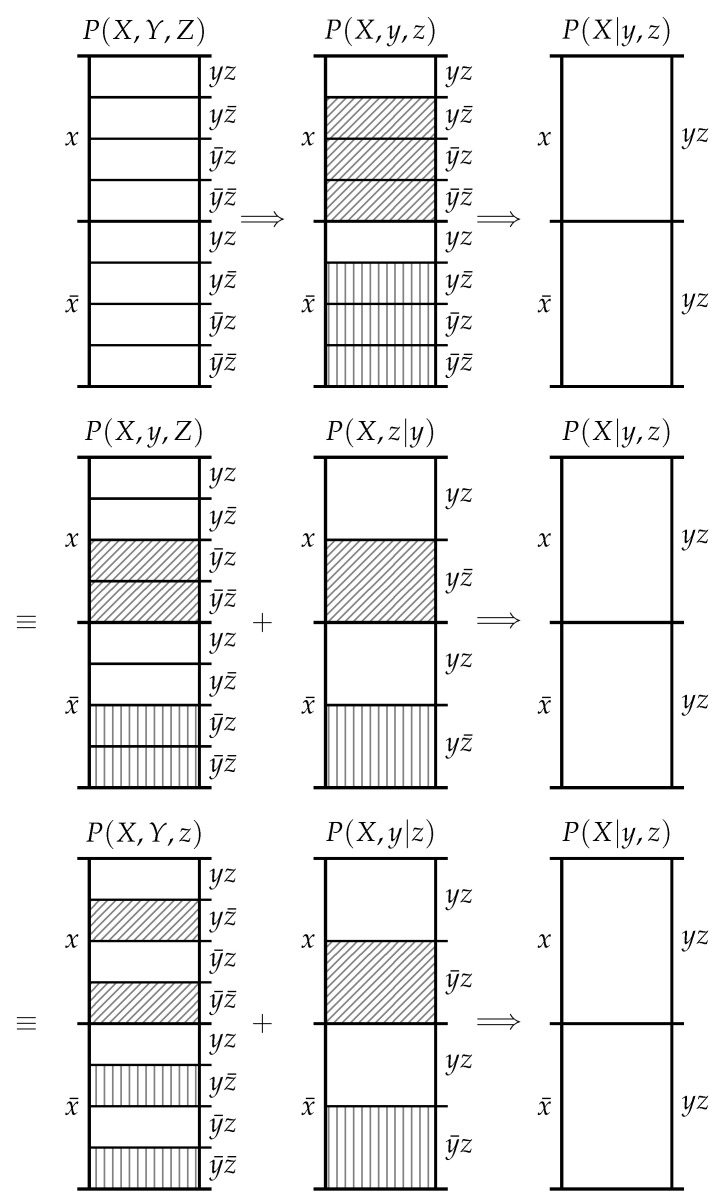
*Top*: *y* and *z* both simultaneously induce probability mass exclusions in P(X) leading directly to P(X|y,z). *Middle*: *y* could induce exclusions in P(X) yielding P(X|y), and then *z* could induce exclusions in P(X|y) leading to P(X|y,z). *Bottom*: the same as the middle, only vice versa in *y* and *z*.

The second observation is that the informative and misinformative exclusions exclusions must individually satisfy the chain rule of probability. As shown in Figure 3, there are three equivalent ways to consider the exclusions induced in P(X) by the events *y* and *z*. Firstly, we could consider the information provided by the joint event yz which excludes the probability mass in P(X) associated with the joint events $y\bar{z}$, $\bar{y}z$ and $\bar{y}\bar{z}$. Secondly, we could first consider the information provided by *y* which excludes the probability mass in P(X) associated with the joint events $\bar{y}z$ and $\bar{y}\bar{z}$, and then subsequently consider the information provided by *z* which excludes the probability mass in P(X|y) associated with the joint event $y\bar{z}$. Thirdly, we could first consider the information provided by *z* which excludes the probability mass in P(X) associated with the joint events $y\bar{z}$ and $\bar{y}\bar{z}$, and then subsequently consider the information provided by *y* which excludes the probability mass in P(X|z) associated with the joint event $\bar{y}z$. Regardless of the chaining, we start with the same p(x) and finish with the same p(x|yz).

**Postulate** **4** (Chain Rule)**.** *The functions $i_{+}\left(y\;\to \;x\right)$ and $i_{-}\left(y\;\to \;x\right)$ satisfy a chain rule; i.e.,*

$$\begin{array}{cc} i_{+}\left(yz\;\to \;x\right) & =i_{+}\left(y\;\to \;x\right)+i_{+}\left(z\;\to \;x|y\right)\\ & =i_{+}\left(z\;\to \;x\right)+i_{+}\left(y\;\to \;x|z\right),\\ i_{-}\left(yz\;\to \;x\right) & =i_{-}\left(y\;\to \;x\right)+i_{-}\left(z\;\to \;x|y\right)\\ & =i_{-}\left(z\;\to \;x\right)+i_{-}\left(y\;\to \;x|z\right), \end{array}$$

*where the conditional notation denotes the same function only with conditional probability as an argument.*

**Theorem** **1.** *The unique functions satisfying Postulates 1–4 are*

(7) $$\begin{array}{ccc} i_{+}\left(y\;\to \;x\right) & =h(y) & =-\log p(y), \end{array}$$

(8) $$\begin{array}{ccc} i_{-}\left(y\;\to \;x\right) & =h(y|x) & =-\log p(y|x). \end{array}$$

By rewriting (7) and (8) in terms of probability mass exclusions, it is easy to verify that Theorem 1 satisfies Postulates 1–4. Perhaps unsurprisingly, this yields a decomposed version of (4),

(9) $$\begin{array}{cc} i_{+}\left(y\;\to \;x\right) & =-\log\left(1-p\left(x,\bar{y}\right)-p\left(\bar{x},\bar{y}\right)\right), \end{array}$$

(10) $$\begin{array}{cc} i_{-}\left(y\;\to \;x\right) & =-\log\left(1-\frac{p(x,\bar{y})}{p(x)}\right). \end{array}$$

Hence, in order to prove Theorem 1 we must demonstrate that (7) and (8) are the unique functions which satisfy Postulates 1–4. This proof is provided in full in Appendix A.

## 5. Discussion

Theorem 1 answers the question posed at the end of Section 3—although there is no one-to-one correspondence between these exclusions and the mutual information, there is a one-to-one correspondence between exclusions and the decomposition

(11) $$\begin{array}{cc} i(x;y) & =i_{+}\left(y\;\to \;x\right)-i_{-}\left(y\;\to \;x\right)\\ & =h(y)-h(y|x). \end{array}$$

It is important to note the directed nature of this decomposition—this equation considers the exclusions induced by *y* with respect to *x*. It is novel that this particular decomposition enables us to uniquely determine the size of the exclusions induced by *y* with respect to *x*, rather than i(x;y)=h(x)−h(x|y), which would not satisfy Postulate 4. Indeed, this latter decomposition is more typically associated with the information provided by *y* about *x* since it reflects the change from the prior p(x) to the posterior p(x|y). Of course, by Theorem 1 this latter decomposition would allow us to uniquely determine the size exclusions induced by *x* with respect to *y*.

There is another important asymmetry which can be seen from (9) and (10). The negative component $i_{-}\left(y\;\to \;x\right)$ depends on the size of *only* the misinformative exclusion while the positive component $i_{+}\left(y\;\to \;x\right)$ depends on the size of *both* the informative and misinformative exclusions. The positive component depends on the total size of the exclusions induced by *y* and hence has no functional dependence on *x*. It quantifies the *specificity* of the event *y*: the less likely the outcome *y* is to occur, the greater the total amount of probability mass excluded by *y* and therefore the greater the potential for *y* to inform about *x*. On the other hand, the negative component quantifies the *ambiguity* of *y* given *x*: the less likely the outcome *y* is to coincide with the outcome *x*, the greater the misinformative probability mass exclusion and therefore the greater the potential for *y* to misinform about *x*. This asymmetry between the components is apparent when considering the two special cases. In the purely informative case where $p(x,\bar{y})=0$, only the positive informational component is non-zero. On the other hand, in the purely misinformative case, both the positive and negative informational components are non-zero, although it is clear that $i_{+}\left(y\;\to \;x\right)<i_{-}\left(y\;\to \;x\right)$ and hence i(x;y)<0.

Let us now consider how this information-theoretic expression (which has a one-to-one correspondence with exclusion) could be utilised to provide an information decomposition that can distinguish between whether events provide the same information or merely the same amount of information. Recall the example from Section 3 where *y* and *z* provide the same amount of information about *x*, and consider this example in terms of the decomposition (11),

(12) $$\begin{array}{cccc} i_{+}\left(y\;\to \;x\right) & =\log_{2}8\;/\;3\;\mathrm{bit}, & \;i_{-}\left(y\;\to \;x\right) & =1\;\mathrm{bit},\\ i_{+}\left(z\;\to \;x\right) & =\log_{2}4\;/\;3\;\mathrm{bit}, & \;i_{-}\left(z\;\to \;x\right) & =0\;\mathrm{bit}. \end{array}$$

In contrast to the mutual information in (5), the decomposition reflects the different ways *y* and *z* provide information through differing exclusions even if they provide the same amount of information. As for how to decompose multivariate information using this decomposition? This is not the subject of this paper—those who are interested in seen an operational definition of shared information based on redundant exclusions should see [12].

## Figures and Tables

**Figure A1:**
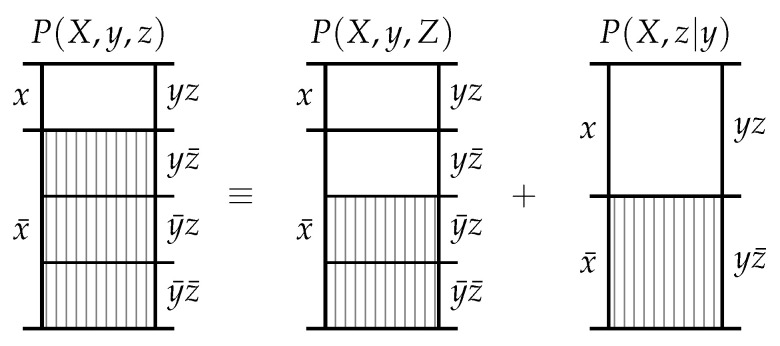
The probability mass diagram associated with (A12). Lemma A2 uses Postulates 3 and 4 to provide a solution for the purely informative case.

**Figure A2:**
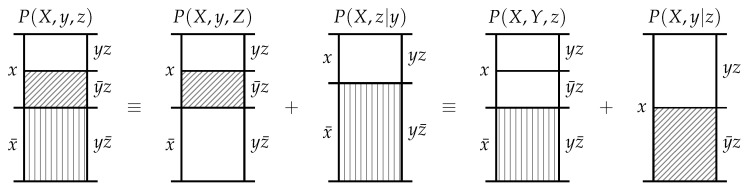
The diagram corresponding to (A14) and (A15). Lemma A3 uses Postulate 4 and Lemma A1 to provide a solution for the purely misinformative case.

**Figure A3:**
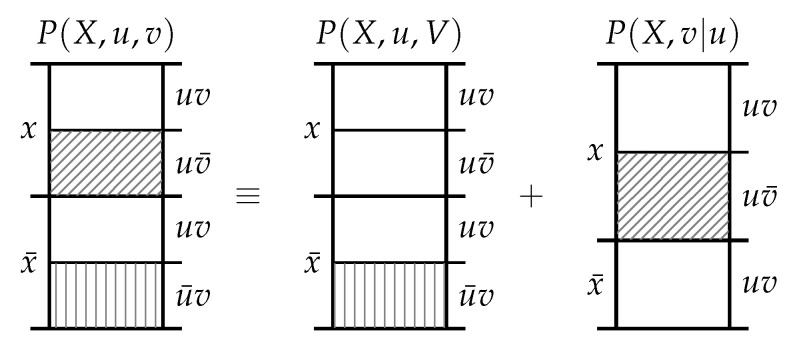
The probability mass diagram associated with (A18) and (A19). Theorem 1 uses Lemmas A2 and A3 to provide a solution to the general case.

## Appendix A

This section contains the proof of Theorem 1. Since it is trivial to verify that (7) and (8) satisfy Postulates 1–4, the proof will focuses on establishing uniqueness. The proof is structured as follows: Lemma A1 considers the functional form required when $p(\bar{x})=0$ and is used in the proof of Lemma A3; Lemmas A2 and A3 consider the purely informative and misinformative special cases respectively; finally, the proof of Theorem 1 brings these two special cases together for the general case.

The proof of Theorem 1 may seem convoluted, however there are two points to be made about this. Firstly, the proof of Lemma A1 is well-known in functional equation theory [13] and is only given for the sake of completeness. (Accepting this substantially reduces the length of the proof.) Secondly, when establishing uniqueness of the two components, we cannot assume that the components share a common base for the logarithm. Specifically, when considering the purely informative case, Lemma A2 shows that the positive component $i_{+}\left(y\;\to \;x\right)$ is a logarithm with same base as the logarithm from Postulate 3, denoted as *b* throughout. On the other hand, considering the purely misinformative case in Lemma A3 demonstrates that the negative component $i_{-}\left(y\;\to \;x\right)$ is a logarithm with base *k* which is greater than or equal to *b*. When combining these in the proof of Theorem 1, it is necessary to show that k=b in order to prove that the components have a common base.

**Lemma** **A1.**
*In the special case where $p(\bar{x})=0$, we have that $i_{+}\left(y\;\to \;x\right)=i_{-}\left(y\;\to \;x\right)=-\log_{k}p\left(y\right)$ with k≥b, where b is the base of the logarithm from Postulate 3.*

**Proof.** That the logarithm is the unique function which satisfies Postulates 2–4 is well-known in functional equation theory [13]; however, for the sake of completeness the proof is given here in full. Since $p(\bar{x})=0$, we have that i(x;y)=0 and hence by Postulate 1, that $i_{+}\left(y\;\to \;x\right)=i_{-}\left(y\;\to \;x\right)$. Furthermore, we also have that $p\left(y\right)=1-p\left(x,\bar{y}\right)$; thus, without loss of generality, we will consider $i_{-}\left(y\;\to \;x\right)$ to be a function of p(y) rather than $p(x,\bar{y})$. As such, let f(m) be our candidate function for $i_{-}\left(y\;\to \;x\right)$ where m=1/p(y). First consider the case where $p(x,\bar{y})=0$, such that m=1. Postulate 4 demands that f(1)=f(1·1)=f(1)+f(1) and hence f(1)=0, i.e., if there is no misinformative exclusion, then the negative informational component should be zero. Now consider the case where $p(x,\bar{y})$ so that *m* is a positive integer greater than 1. If *r* is an arbitrary positive integer, then $2^{r}$ lies somewhere between two powers of *m*, i.e., there exists a positive integer *n* such that

(A1) $$m^{n}\leq 2^{r}<m^{n+1}.$$

So long as the base *k* is greater than 1, the logarithm is a monotonically increasing function, thus

(A2) $$\log_{k}m^{n}\leq \log_{k}2^{r}<\log_{k}m^{n+1},$$

or equivalently,

(A3) $$\frac{n}{r}\leq \frac{\log_{k}2}{\log_{k}m}<\frac{n+1}{r}.$$

By Postulate 2, f(m) is a monotonically increasing function of *m*, hence applying it to (A1) yields

(A4) $$f\left(m^{n}\right)\leq f\left(2^{r}\right)<f\left(m^{n+1}\right).$$

Note that, by Postulate 4 and mathematical induction, it is trivial to verify that

(A5) $$f\left(m^{n}\right)=n\cdot f\left(m\right).$$

Hence, by (A4) and (A5), we have that

(A6) $$\frac{n}{r}\leq \frac{f(2)}{f(m)}<\frac{n+1}{r}.$$

Now, (A3) and (A6) have the same bounds, hence

(A7) $$\left|\frac{\log_{k}2}{\log_{k}m}-\frac{f(2)}{f(m)}\right|\leq \frac{1}{r}.$$

Since *m* is fixed and *r* is arbitrary, let r→∞. Then, by the squeeze theorem, we get that

(A8) $$\begin{array}{cc} \frac{\log_{k}2}{\log_{k}m} & =\frac{f(2)}{f(m)}, \end{array}$$

and here

(A9) $$\begin{array}{cc} f(m) & =\log_{k}m. \end{array}$$

Now consider the case where $p(x,\bar{y})$ so that *m* is a rational number; in particular, let m=s/r where *s* and *r* are positive integers. By Postulate 4,

(A10) f(s)=f(s/r·r)=f(s/r)+f(r)=f(m)+f(r).

Thus, combining (A9) and (A10), we get that

(A11) $$f\left(m\right)\;=\;f\left(s\right)-f\left(r\right)=\log_{k}\left(s\right)-\log_{k}\left(r\right)=\log_{k}m.$$

Now consider the case where $p(x,\bar{y})$ such that *m* is a real number. By Postulate 2, the function (A11) is the unique solution, and hence, $i_{+}\left(y\;\to \;x\right)=i_{-}\left(y\;\to \;x\right)=-\log_{k}p\left(y\right)$. Finally, to show that k≥b, consider an event z=y. By Postulate 3, $i_{+}\left(y\;\to \;z\right)=-\log_{b}p\left(y\right)$. Furthermore, since $p\left(\bar{z},\bar{y}\right)\geq p\left(\bar{x},\bar{y}\right)=0$, by Postulate 2, $i_{+}\left(y\;\to \;z\right)\geq i_{+}\left(y\;\to \;x\right)$. Thus, $-\log_{b}p\left(y\right)\geq -\log_{k}p\left(y\right)$, and hence k≥b. □

**Lemma** **A2.**
*In the purely informative case where $p(x,\bar{y})=0$, we have that $i_{+}\left(y\;\to \;x\right)=-\log_{b}p\left(y\right)$ and $i_{-}\left(y\;\to \;x\right)=0$, where b is the base of the logarithm from Postulate 3.*

**Proof.** Consider an event *z* such that x=yz and $\bar{x}=\left\{y\bar{z},\bar{y}z,\bar{y}\bar{z}\right\}$. By Postulate 4,

(A12) $$i_{+}\left(yz\;\to \;x\right)=i_{+}\left(y\;\to \;x\right)+i_{+}\left(z\;\to \;x|y\right),$$

as depicted in Figure A1. By Postulate 3, $i_{+}\left(yz\;\to \;x\right)=h\left(x\right)$ and $i_{+}\left(z\;\to \;x|y\right)=h\left(x|y\right)$, where the latter equality follows from the equivalence of the events *x* and *z* given *y*. Furthermore, since $p(x,\bar{y})=0$, we have that p(x,y)=p(x), and hence that p(y|x)=1. Thus, from (A12), we have that

(A13) $$\begin{array}{cc} i_{+}\left(y\;\to \;x\right) & =h(x)-h(x|y)\\ & =h(y)-h(y|x)\\ & =h(y). \end{array}$$

Finally, by Postulate 1, $i_{-}\left(y\;\to \;x\right)=0$. □

**Lemma** **A3.**
*In the purely misinformative case where $p(\bar{x},\bar{y})=0$, we have that $i_{+}\left(y\;\to \;x\right)=h\left(y\right)-h\left(y|x\right)-\log_{k}p\left(y|x\right)$ and $i_{-}\left(y\;\to \;x\right)=-\log_{k}p\left(y|x\right)$ with k≥b, where b is the base of the logarithm from Postulate 3.*

**Proof.** Consider an event z=x. By Postulate 4,

(A14) $$\begin{array}{cc} i_{+}\left(yz\;\to \;x\right) & =i_{+}\left(y\;\to \;x\right)+i_{+}\left(z\;\to \;x|y\right)\\ & =i_{+}\left(z\;\to \;x\right)+i_{+}\left(y\;\to \;x|z\right), \end{array}$$

(A15) $$\begin{array}{cc} i_{-}\left(yz\;\to \;x\right) & =i_{-}\left(y\;\to \;x\right)+i_{-}\left(z\;\to \;x|y\right)\\ & =i_{-}\left(z\;\to \;x\right)+i_{-}\left(y\;\to \;x|z\right), \end{array}$$

as depicted in Figure A2. Since z=x, by Postulate 3, $i_{+}\left(z\;\to \;x\right)=h\left(x\right)$, $i_{-}\left(z\;\to \;x\right)=0$, $i_{+}\left(z\;\to \;x|y\right)=h\left(x|y\right)$ and $i_{-}\left(z\;\to \;x|y\right)=0$. Furthermore, since $p(\bar{x}|z)=0$, by Lemma A1, $i_{+}\left(y\;\to \;x|z\right)=i_{-}\left(y\;\to \;x|z\right)=-\log_{k}p\left(y|z\right)=-\log_{k}p\left(y|x\right)$, hence, from (A14) and (A15), we get that

(A16) $$\begin{array}{cc} i_{+}\left(y\;\to \;x\right) & =h\left(x\right)-h\left(x|y\right)-\log_{k}p\left(y|x\right)\\ & =h\left(y\right)-h\left(y|x\right)-\log_{k}p\left(y|x\right), \end{array}$$

(A17) $$\begin{array}{cc} i_{-}\left(y\;\to \;x\right) & =-\log_{k}p\left(y|x\right), \end{array}$$

as required. □

**Proof** **of** **Theorem** **1.** In the general case, both $p(\bar{x},\bar{y})$ and $p(x,\bar{y})$ are non-zero. Consider two events, *u* and *v*, such that y=uv, $p(x,\bar{u})=0$ and $p(\bar{x},\bar{v})=0$. By Postulate 4,

(A18) $$\begin{array}{cc} i_{+}\left(y\;\to \;x\right)=i_{+}\left(uv\;\to \;x\right) & =i_{+}\left(u\;\to \;x\right)+i_{+}\left(v\;\to \;x|u\right), \end{array}$$

(A19) $$\begin{array}{cc} i_{-}\left(y\;\to \;x\right)=i_{-}\left(uv\;\to \;x\right) & =i_{-}\left(u\;\to \;x\right)+i_{-}\left(v\;\to \;x|u\right), \end{array}$$

as depicted in Figure A3. Since $p(x,\bar{u})=0$, by Lemma A2, $i_{+}\left(u\;\to \;x\right)=h\left(u\right)$ and $i_{-}\left(u\;\to \;x\right)=0$; furthermore, we also have that p(x)=p(x,u), and hence p(v|xu)=p(uv|x). In addition, since $p(\bar{x},\bar{v}|u)=0$, by Lemma A3, we have that $i_{+}\left(v\;\to \;x|u\right)=h\left(v|u\right)+h\left(v|xu\right)-\log_{k}p\left(v|xu\right)$ and $i_{-}\left(v\;\to \;x|u\right)=-\log_{k}p\left(v|xu\right)$ where k≥b. Therefore, by (A18) and (A19),

(A20) $$\begin{array}{cc} i_{+}\left(y\;\to \;x\right) & =h\left(u\right)+h\left(v|u\right)-h\left(v|xu\right)-\log_{k}p\left(v|xu\right)\\ & =h\left(y\right)-h\left(y|x\right)-\log_{k}p\left(y|x\right), \end{array}$$

(A21) $$\begin{array}{cc} i_{-}\left(y\;\to \;x\right) & =-\log_{k}p\left(v|xu\right)\\ & =-\log_{k}p\left(y|x\right). \end{array}$$

Finally, since Postulate 1 requires that $i_{+}\left(y\;\to \;x\right)\geq 0$, we have that $h\left(y\right)-h\left(y|x\right)-\log_{k}p\left(y|x\right)\geq 0$, or equivalently,

(A22) $$\log_{b}p\left(y\right)\leq \left(1-\frac{1}{\log_{b}k}\right)\log_{b}p\left(y|x\right).$$

This must hold for all p(y) and p(y|x), which is only true in general for b≥k. Hence, k=b and therefore

(A23) $$\begin{array}{cc} i_{+}\left(y\;\to \;x\right) & =h\left(y\right)-h\left(y|x\right)-\log_{b}p\left(y|x\right)\\ & =h(y), \end{array}$$

(A24) $$\begin{array}{cc} i_{-}\left(y\;\to \;x\right) & =-\log_{b}p\left(y|x\right)\\ & =h(y|x).\;□ \end{array}$$

**Corollary** **A1.**
*The conditional decomposition of the information provided by y about x given z is given by*

(A25) $$\begin{array}{ccc} i_{+}\left(y\;\to \;x|z\right) & =h(y|z) & =-\log p(y|z), \end{array}$$

(A26) $$\begin{array}{ccc} i_{-}\left(y\;\to \;x|z\right) & =h(y|xz) & =-\log p(y|xz). \end{array}$$

**Proof.** Follows trivially using conditional distributions. □

**Corollary** **A2.**
*The joint decomposition of the information provided by y and z about x is given by*

(A27) $$\begin{array}{ccc} i_{+}\left(yz\;\to \;x\right) & =h(yz) & =-\log p(yz), \end{array}$$

(A28) $$\begin{array}{ccc} i_{-}\left(yz\;\to \;x\right) & =h(yz|x) & =-\log p(yz|x). \end{array}$$

*The joint decomposition of the information provided by y about x and z is given by*

(A29) $$\begin{array}{ccc} i_{+}\left(y\;\to \;xz\right) & =h(y) & =-\log p(y), \end{array}$$

(A30) $$\begin{array}{ccc} i_{-}\left(y\;\to \;xz\right) & =h(y|xz) & =-\log p(y|xz). \end{array}$$

**Proof.** Follows trivially using joint distributions. □

**Corollary** **A3.**
*We have the following three identities,*

(A31) $$\begin{array}{cc} i_{+}\left(y\;\to \;x\right) & =i_{+}\left(y\;\to \;z\right), \end{array}$$

(A32) $$\begin{array}{cc} i_{+}\left(y\;\to \;x|z\right) & =i_{-}\left(y\;\to \;z\right), \end{array}$$

(A33) $$\begin{array}{cc} i_{-}\left(y\;\to \;x|z\right) & =i_{-}\left(y\;\to \;xz\right). \end{array}$$

**Proof.** The identity (A31) follows from (7), while (A32) follows from (8) and (A25); finally, (A33) follows from (A26) and (A30). □

Finally, it is not true that the components satisfy a target chain rule. That is, in general the following relation $i_{+}\left(y\;\to \;xz\right)=i_{+}\left(y\;\to \;x\right)+i_{+}\left(y\;\to \;z|x\right)$ does not hold, nor does $i_{-}\left(y\;\to \;xz\right)=i_{-}\left(y\;\to \;x\right)+i_{-}\left(y\;\to \;z|x\right)$. However, the mutual information must satisfy a chain rule over target events. Thus, it is interesting to observe how the target chain rule for mutual information arises in terms of exclusions. The key observation is that the positive informational component provided by *y* about *z* given *x* equals the negative informational component provided by *y* about *z*, as per (A32).

**Corollary** **A4.**
*The information provided by y about x and z satisfies the following chain rule,*

(A34) $$\begin{array}{cc} i(y\;\to \;xz) & =i(y\;\to \;x)+i(y\;\to \;z|x). \end{array}$$

**Proof.** Starting from the joint decomposition (A29) and (A30). By the identities (A31) and (A33), we get that

(A35) $$\begin{array}{cc} i(y;xz) & =i_{+}\left(y\;\to \;xz\right)-i_{-}\left(y\;\to \;xz\right),\\ & =i_{+}\left(y\;\to \;x\right)-i_{-}\left(y\;\to \;z|x\right), \end{array}$$

Then, by identity (A32), and recomposition, we get that

(A36) $$\begin{array}{cc} i(y;xz) & =i_{+}\left(y\;\to \;x\right)-i_{-}\left(y\;\to \;x\right)\\ & \;+i_{-}\left(y\;\to \;x\right)-i_{-}\left(y\;\to \;z|x\right),\\ & \begin{array}{c} =i_{+}\left(y\;\to \;x\right)-i_{-}\left(y\;\to \;x\right)\\ \;+i_{+}\left(y\;\to \;z|x\right)-i_{-}\left(y\;\to \;z|x\right), \end{array}\\ & =i(y;x)+i(y;z|x).\;□ \end{array}$$
